# Diffusion of nanoparticles in heterogeneous hydrogels as vitreous humour in vitro substitutes

**DOI:** 10.1038/s41598-024-68267-0

**Published:** 2024-07-29

**Authors:** Moira Lorenzo Lopez, Victoria R. Kearns, Judith M. Curran, Eann A. Patterson

**Affiliations:** 1 School of Engineering, University of Liverpool, Liverpool, L69 3BX UK; 2 Department of Eye and Vision Science, University of Liverpool, Liverpool, L7 8TX UK

**Keywords:** Retinal diseases, In vitro models, Hydrogels, Nanomedicine, Drug delivery, Label-free tracking, Drug delivery, Drug delivery, Retinal diseases

## Abstract

Nanomedicine has the potential to increase the biostability of drugs to treat retinal diseases, improving their performance and decreasing the required number of intravitreal injections. However, accurate pharmacokinetic studies of these nanoparticle-drug conjugates, nanoparticle motion across the vitreous humour and interaction with the retinal cell layers still need to be investigated. Existing nanoparticle tracking techniques require fluorescent labels, which can impact cytotoxicity, nanoparticles’ motion, protein interactions, and cell internalization. In this study, a real-time label-free tracking technology, for single nanoparticles in an optical microscope based on the optical phenomena of caustics, was used to characterise the diffusion of nanoparticles in agar-hyaluronic acid hydrogels, previously validated as vitreous humour substitutes for in vitro models. The results demonstrated that the diffusion of nanoparticles through these hydrogels was heterogeneous, and that nanoparticle size had an important role in nanoparticle distribution across and within in vitro vitreous substitutes. These findings suggest that nanoparticle diameter is a critical parameter for designing novel therapeutics for retinal diseases. Moreover, nanoparticle charge did not affect nanoparticle diffusion or distribution in these synthetic hydrogels. The use of caustics in optical microscopy has been demonstrated to be a reproducible, inexpensive technique for screening novel therapeutics in eye in vitro models.

## Introduction

Vision impairment creates an economic burden of £25 billion in the UK alone^1^, and diseases affecting the retina, such as macular degeneration (MD) and diabetic retinopathy (DR), affect more than 12 million people globally^2^. These numbers will grow as a result of an increase in life expectancy worldwide. Considering that current treatments for retinal diseases involve monthly or bimonthly intravitreal injections of anti-vascular endothelium growth factor (anti-VEGF) agents and corticoids, there is an urgent need to optimize drug delivery and increase the sustained efficacy of treatment in the retina^3,4^ so that the patient’s quality of life can be increased and the economic burden on health care systems can be reduced.

Numerous advancements have been noted in ocular drug delivery systems with the goal of optimising intraocular drug delivery^5,6^. Among these, the conjugation of first-line approved drugs with functionalized nanoparticles (NPs) has the potential to increase bioavailability in the posterior chamber, increase the concentration in the retinal cell layer, and minimize side effects by decreasing the number of intravitreal injections^7,8^. To optimize the drug delivery profile of these NP-drug conjugates, experimental models are required to characterize the diffusion of nanoparticle delivery systems across the vitreous humour (VH).

The well-characterized drawbacks of in vivo animal testing, such as ethical concerns and poor representation of human models make the use of small animals, to test retinal-targeted drugs, inadequate and a waste of resources^9,10^. The utilization of cadaveric eyes for ex vivo analysis presents issues of stability once the vitreous humour is detached from the retina for analysis, at the same time, the cadaveric nature will affect the stability of the biomechanical and biochemical properties, affecting the accuracy and replicability of the results^11,12^. The development of a pre-clinical in vitro model of the eye reduces the need for living or cadaveric donors, could improve the stability of the vitreous-like biomechanical properties, could increase the replicability of the results, and could lower the economic cost of screening novel therapeutics.

The first biological barrier that a NP-drug conjugate faces following intravitreal injection is the vitreous humour, hence significant effort has been made to replicate its biomechanical properties to evaluate pharmacokinetics, to optimize dosage and to formulate the design of novel therapeutics as well as to characterize the real concentration that reaches the targeted retinal cell layer^13–15^. Despite this effort, there is still no clear understanding of nanoparticle diffusion and distribution through the vitreous humour for back-of-the-eye in vitro pre-clinical models^16^.

In 2020, Thakur et al., validated previously studied agar-hyaluronic acid hydrogels as vitreous humour substitutes for in vitro drug delivery screening^13,17^. The authors evaluated the rheological properties of the hydrogels and compared them to bovine-extracted vitreous humour, the data showed that the hydrogel with the lowest viscosity (LV) was the closest to mimicking the biomechanical properties of bovine vitreous humour^14^. Moreover, for polystyrene nanoparticles that had been fluorescently tagged, their data suggested that the rate of diffusion in low and medium viscosity synthetic hydrogels was the same as that in vitreous humour.

However, nanoparticle-labelling has several limitations; including photobleaching and the requirement for meticulous sample preparation, which translates into time-consuming and expensive practices^18^. In particular, when used for in vitro purposes, we need to take into consideration that some dyes exhibit cytotoxicity due to the production of free radicals after excitation or phototoxicity induced by the laser or light beams used to detect them, affecting the overall aim of the test^19^. Moreover, a recent study has stated that the use of different labelling methods will have a direct impact on the diffusive behavior of particles, creating a large burden on the standardization, accuracy, and replicability of in vitro models, where the results are dependent on the nature of the fluorescent dye^20^.

**Figure 1:**
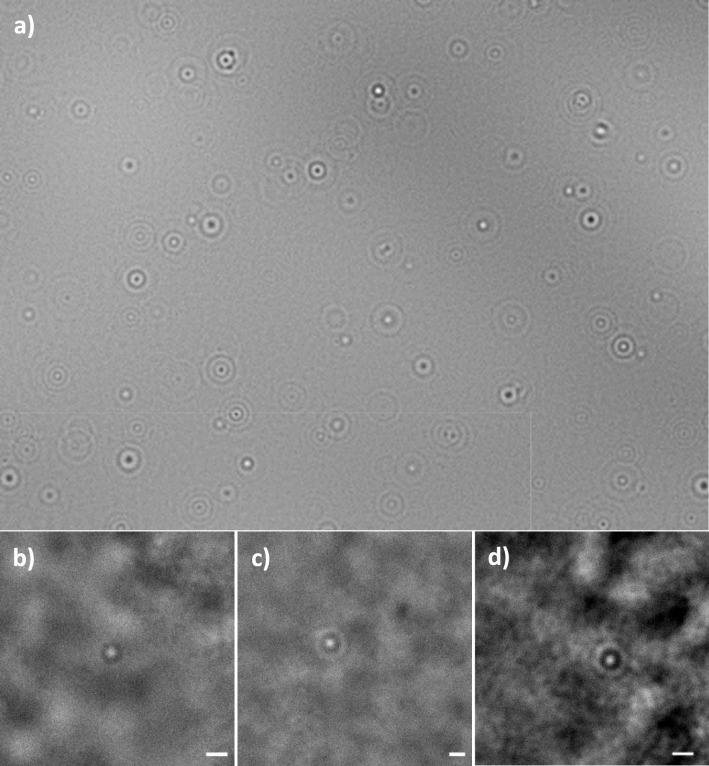
Label-free visualization of nanoparticles with caustics optical microscopy technique, visible through the creation of optical signatures. (**a**) Optical signatures of 100 nm diameter gold nanoparticles in a 50% glycerol deionized water solution (v/v); (**b**) Single 50 nm diameter gold nanoparticle in low viscous hydrogel; (**c**) Single 100 nm diameter gold nanoparticle in medium viscous hydrogel and (**d**) Single 100 nm diameter nanoparticle in a high viscous hydrogel. Scale bar: 20 µm.

Single-particle tracking has emerged as a potential tool to better understand particle diffusion and interactions with hydrogels and other soft matter systems^21^. This is mainly due to the possibility of tracking nanoparticles in real-time in the media of interest, as opposed to other high-resolution (nanoscale) techniques, such as electronic microscopy^22^. Recently, Foreman and Tran-Ba used this technique to analyze nanoparticle diffusive behavior in polymer solutions, revealing the size-dependent and steric-dependent behavior of carboxylic fluorescently modified nanoparticles and quantum dots^23^. However, this label-based technique can affect the physicochemical-surface properties of nanoparticles, impacting their dynamics and distribution through the hydrogels. Considering the previously highlighted disadvantages of fluorescence microscopy, and the clinical motivations of our experimental design, we have used a label-free, single-particle tracking technique that enables the visualization and localization of nanoparticles in real-time. This technique exploits the naturally-occurring optical phenomenon of caustics, which is defined as the three-dimensional envelopes of bright light and corresponding shadows created by the refraction (or reflection) of light when it is transmitted (or reflected) by a curved surface^24^. This phenomenon was applied in a standard, inverted light optical microscope, by increasing the coherence of the source of light^25^, generating optical signatures several orders of magnitude larger than the particle’s actual size (Fig. 1), thus allowing the nanoparticles to be visualized and tracked without fluorescent dye, in a fast and cost-effective manner^26^, see for example Fig. 1.

Previous studies investigated the motion of metallic and polymeric nanoparticles in simple fluids^26,27^ and in this study gold nanoparticles, with diameters from 50 to 200 nm, were used as these nanoparticles have been widely studied and characterized in the literature^28,29^. The motion of the particles through hydrogels, which were synthesized with three concentration levels of agar and hyaluronic acid resulting three levels of viscosity, was characterized with the label-free technique in an inverted optical microscope. The motion of the caustic signature from individual nanoparticles were captured by a video camera during the random walk of the particle induced by Brownian motion. Measurements of the random walk were used to characterize the diffusion behaviour of the particles in the different gels. The initial results, based on tracking six independent particles in each hydrogel, revealed the heterogeneous nature of the hydrogels, which contained regions of low and medium viscosity, thus affecting nanoparticle diffusion and distribution. A second study was performed in which thirty randomly selected nanoparticles were tracked in each hydrogel to allow the diffusion behaviour to confidently characterized using their diffusion coefficients and the minimum area enclosing their random walks during a given time period. The results demonstrate that both the diameter of the nanoparticle and the structure of the hydrogel affect the diffusion behaviour and that the behaviour in low and medium viscosity hydrogels mimics that in porcine ex vivo vitreous humour published previously better than the behaviour in high viscosity hydrogels. This paper shows the potential of this label-free technique for nanoparticle tracking in complex heterogeneous polymeric hydrogels.

## Materials and methods

### Materials

#### Gold nanoparticles

50 nm (− 34.5 mV), 100 nm (-32.7 mV) and 200 nm diameter (-31 mV) spherical citrate-capped gold nanoparticles (AuNPs) were purchased from BBI Solutions (Crumlin, UK), and 100 nm diameter (28.7 mV) branched polyethylenimine (BPEI) capped AuNPs were obtained from NanoComposix. These nanoparticle stock solutions (10^−2^ mg/mL), were diluted in ultra-pure deionized water to obtain the desired constant concentration of 10^–4^ mg/mL when added to the hydrogels, see Supplementary Table 1 (in supplementary information).

#### Microscope

An Axio Observer.Z1 m (Carl Zeiss, DE) inverted optical microscope was used to create optical signatures or caustics that enabled the identification and tracking of gold nanoparticles in the viscous media. This was possible through an increase in the coherence of the light (source: halogen lamp) by closing the aperture of the condenser to its minimum (1 mm), with the field diaphragm fully open, adding a green interference filter (Olympus, JP, centred on 550 nm, 45 nm bandwidth) and adjusting the microscope for Köhler illumination by adjusting the condenser height to where the light throughput was maximised; following the methodology described by Patterson and Whelan^26^. The microscope was also equipped with a stage-top incubation system (Incubator PM S1, Heating Insert P S1, Temp and CO_2_ module S1, Carl Zeiss, DE), which was used to maintain the samples at 34 °C corresponding to the physiological mid-vitreous temperature, and is within the temperature range for characterising ocular endotamponades^30,31^.

### Methods

#### Agar-hyaluronic acid hydrogel synthesis

Hydrogels were synthesised following the protocol described by Thakur et al.^14^. Three hydrogels with different viscosities were synthesised by adding different concentrations of agar and hyaluronic acid (high molecular weight) to 1X boiling phosphate buffer saline (PBS). For the high viscous (HV) hydrogel, hyaluronic acid was at a concentration of 5 mg/mL and agar at 4 mg/mL; for the medium viscous (MV) hydrogel, hyaluronic acid was at a concentration of 2.21 mg/mL and agar at 1.8 mg/mL; and for the low viscous (LV) hydrogel, hyaluronic acid was at a concentration of 0.7 mg/mL and agar at 0.95 mg/mL. The three solutions were magnetically stirred (500–1000 rpm) at 100˚C for 1 h, after which the gels were cooled down at room temperature overnight before being characterized or used for NP tracking.

#### Cryo-SEM

Vitreous humour extracted from porcine eyes (from a local abattoir) and the synthetic hydrogels were analysed under cryo-SEM using a Tescan FIB SEM S8000G microscope. The hydrogels were deposited into planchets and frozen in a nitrogen slush (− 210 °C), these were transferred into the preparation chamber under vacuum. Samples were stabilised in the preparation chamber for 5 min then fractured with a built-in scalpel and sublimated under vacuum for 5 min. Prior to analysis these were coated with platinum. Imaging was carried at 1.5 keV and 15 pA.

#### Diffusion of gold nanoparticles in hydrogels

The diffusion coefficient of positively-charged 100 nm AuNP and 50 nm, 100 nm and 200 nm diameter negatively-charged AuNP was evaluated at 34 °C. Once the AuNPs were added to the hydrogels, the samples were vortexed (with a Vortex-Fisherbrand® mixer, UK) for 15 s and ultrasonicated (with a S-Series Heated Ultrasonic Bath Fisherbrand®, UK) for 1 min before aliquoting a volume of 50 µL onto a microscope slide (76 × 26 mm and 0.2 mm cavity depth). This resulted in a random distribution of individual particles. The samples were incubated on the microscope stage-top incubation system at 34 °C for 10 min prior to being analysed under an inverted optical microscope, to ensure a consistent temperature throughout the sample. Videos were recorded using a × 40 objective and a monochromatic camera (Axiocam305, Carl Zeiss, DE) with a spatial resolution of 0.086 µm^2^ for one pixel and a temporal resolution of 40 fps. A total of 30 nanoparticles were tracked for each nanoparticle size in each of the hydrogels, in order to randomise the nanoparticle distribution across the hydrogels. For the preliminary quantitative analysis and investigation of the nanoparticle surface charge, six nanoparticles, randomly selected from the field of view, were tracked for each set of conditions and the mean value of diffusion coefficient calculated. The same process was repeated in deionized water as a control.

#### Data analysis

An assumption of Brownian motion for a random walk of particles in a suspended fluid leads to the following definition of the diffusion coefficient (*D*):

1 $$D= \frac{{k}_{b} T}{4d\pi nr}$$

In the Eq. (1) *k*_*b*_ is the Boltzmann constant, *T* is the temperature, *d* is the dimensionality (2D in this case), *n* is the viscosity of the diffusive media and *r* is the radius of the particle. This is known as the Stokes–Einstein relation because it combines Stokes’ Law and Einstein’s description of diffusion. Particle motion has been widely characterised by the mean squared displacement (MSD) of the particle and, considering a two-dimensional system, is expressed as^32,33^:

2 $$MSD=\frac{1}{N-n}{\sum_{i=0}^{N-n} (({x}_{i+n}-{x}_{i})}^{2}+{({y}_{i+n}-{y}_{i})}^{2})$$

where *N* is the total data points, *x* and *y* correspond to the particle coordinates for each *i*^*th*^ step in *n* total frames and hence can be measured experimentally.

Videos with a minimum of 50 frames and a maximum of 100 frames, depending on the speed of a particle and its motion perpendicular to the focal plane of the microscope, were recorded and analysed using the ImageJ software plugin TrackMate^34^. This provided information on the *x* and *y* positions of the nanoparticle for each frame. These coordinates provided the path of the NPs over time, from which values of their mean squared displacement (MSD) and hence the experimental diffusion coefficient were determined, as there is a linear relation between the mean squared displacement and the diffusion coefficient (*D*), for Brownian motion, for a specific time lag ($\Delta t):$

3 $$D= \frac{MSD}{2d\Delta t}$$

To better characterise the nanoparticle distribution through the hydrogels, the area containing the path of a nanoparticle was evaluated and compared to the values of their diffusion coefficient. The path of a nanoparticle through 50 frames was plotted and all the points belonging to it were enclosed in the smallest possible polygon defined by a convex hull, which was found using a geometric algorithm^35^.

## Results and discussion

**Figure 2:**
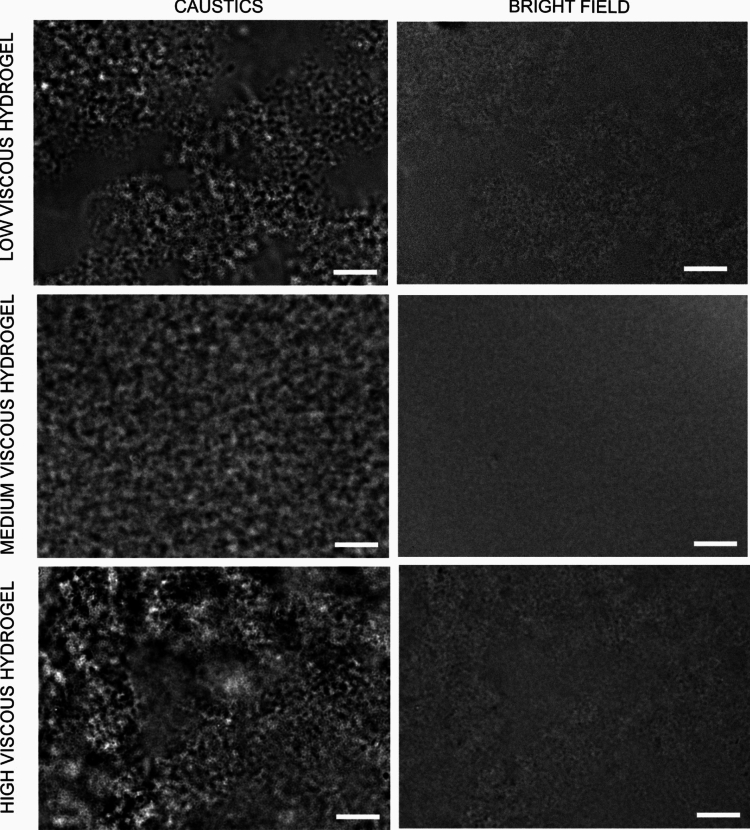
Qualitative characterization of agar-hyaluronic acid hydrogels visualized using the caustics optical technique (left) and normal bright field (right), images obtained from the same sample area, for each of the different viscous hydrogels, with an inverted optical microscope. Scale bar: 20 µm.

Initial qualitative visualization of the agar-hyaluronic acid hydrogels, with the microscope set up to observe caustics, revealed a detailed heterogeneous nature of the synthetic hydrogels (Fig. 2). This was confirmed by analysis of nanoparticle motion, in which the nanoparticles were found to behave differently in different local environments within the hydrogels. NPs were found to diffuse faster in environments with a high-water content (aqueous phase) and slower when interacting with the agar (gel phase) (videos attached in supplementary information).

**Figure 3:**
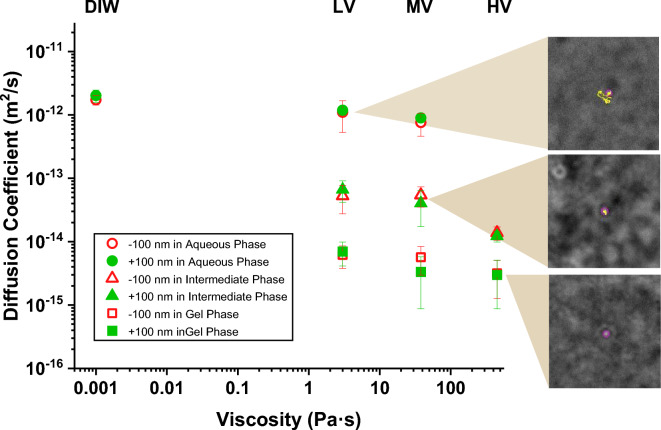
Diffusion coefficient values of 100 nm diameter gold nanoparticles (AuNP) positively charged (green) and negatively charged (red) in deionized water (DIW = 0.001 Pa·s) and in the agar hyaluronic acid hydrogels: low viscous (LV = 1.4 Pa·s), medium viscous (MV = 10 Pa·s) and high viscous (HV = 180 Pa·s) at the physiological eye temperature of 34˚C. Showing the diffusion values for each hydrogel phase; aqueous phase (circles), intermediate phase (triangles), and gel phase (squares). Error bars correspond to ± 1 standard deviation. Images represent the AuNP trails of motion in the three different hydrogel phases: aqueous, intermediate and gel (from top to bottom); in the three hydrogels: LV, MV, and HV (from top to bottom).

To validate these qualitative findings, we performed a preliminary quantitative analysis by tracking six independent nanoparticles, using the label-free caustics optical technique, in low, medium, and high viscosity solutions that were designed to represent the qualitatively characterized environments, identified from Fig. 2 and described above, i.e., an aqueous phase, gel phase and intermediate phase. The experimental diffusion coefficients of the nanoparticles in each local environment are plotted against the static bulk viscosity for each hydrogel (characterization in supplementary information) and compared to that of deionized water, which was used as a control, in Fig. 3. To investigate the effect of nanoparticle surface charge on nanoparticle diffusion across the synthetic hydrogels, citrate-capped (negative) and branched polyethyleneimine (BPEI)-capped (positive) 100 nm diameter gold nanoparticles were tracked as described above using the caustic technique.

Considering that the presence of hyaluronic acid will give the hydrogels a partial negative charge, a difference in diffusion would be expected for differently charged nanoparticles^36^. However, the results in Fig. 3 show no definitive difference between the diffusion of positively (closed green symbols) and negatively (open red symbols) charged AuNPs across any of the gels. This was explained by the low zeta potential values of the hydrogels, characterizing these as neutral (Table 1), and the low nanoparticle concentration present in the hydrogels. More interestingly the values of diffusion coefficient were found to range from 10^–12^ to 10^–15^ m^2^/s for both, negatively and positively-charged 100 nm diameter AuNPs in the low and medium viscous hydrogels (Fig. 3).

**Table 1:** Agar-hyaluronic acid hydrogels characterization.

Hydrogel	Agar (mg/mL)	Hyaluronic acid (mg/mL)	Zeta potential (mV)	Static viscosity (Pa·s)	G’ (Pa)	G’’ (Pa)	pH
LV A-HA	0.95	0.70	− 6.7	3	1.03	0.15	7.23
MV A-HA	1.80	2.21	3.0	38	6.81	0.83	7.27
HV A-HA	4.00	5.00	− 3.0	450	136.1	16.58	7.20
VH (PORCINE)	–	0.16^37^	− 9.9	1	0.35	0.15	7.35

Agar and hyaluronic acid concentrations were determined as by Thakur et al. Viscosity and rheological analysis were performed at 34 °C via in-house characterization.

**Figure 4:**
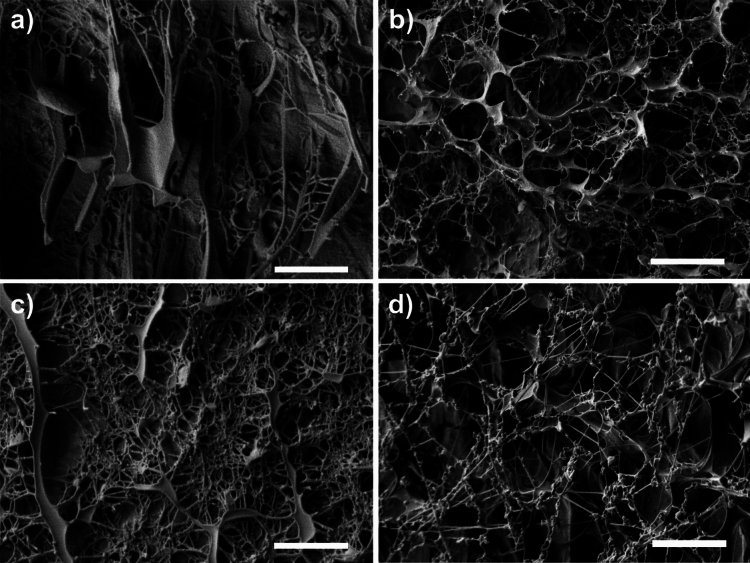
Cryo-SEM images of (**a**) Low viscous AHA hydrogel; (**b**) Medium viscous AHA hydrogel; (**c**) High viscous AHA hydrogel; and (**d**) Porcine Vitreous Humour. Scale bar 2 µm.

Deionized water was used as a control with a viscosity three orders of magnitude less than the hydrogel with lowest viscosity (approximately 0.001 Pa·s compared to 3 Pa·s). The medium and high viscosity hydrogels had viscosities one and two orders of magnitude higher than the low viscosity hydrogel, (i.e., approximately 40 and 400 Pa·s respectively). In the low and medium viscosity hydrogels, some of the tracked particles behaved in the same manner as those in the deionised water and their diffusion coefficients values were similar (approximately 10^–12^ m^2^/s). These nanoparticles had a random walk which led them away from the location at which they were first observed, as shown in the top inset in Fig. 3 (aqueous phase). However, some particles moved randomly around a single location, essentially dancing on the spot (see bottom inset in Fig. 3); these nanoparticles had values for their diffusion coefficient of about 10^–15^ m^2^/s (gel phase). This behaviour occurred in all three hydrogels with the diffusion coefficient decreasing in value with increasing viscosity, implying that the high viscosity inhibited the motion of the particles and constrained them from moving away from a given location. The observation that both of these types of behaviour, i.e. wandering away from a location and dancing on the spot, occurred the same in low and medium viscosity hydrogels implies that these hydrogels were heterogeneous with some zones that were largely aqueous solution and others that were largely gel, consisting of a polymeric matrix that inhibited nanoparticle motion. There is also evidence of nanoparticles diffusing in intermediate zones with properties that lie somewhere between those of the gel and aqueous solution, with diffusive values around 10^–14^ m^2^/s, see middle inset in Fig. 3, which shows a typical path of particles attempting to move away from a location but repeatedly returning to it (intermediate phase). These different behaviours within the low and medium viscosity hydrogels were not observed in the high viscosity hydrogel which implied that it was a largely homogeneous gel, with a mesh size, hence an aqueous phase smaller than the diameter of tracked nanoparticles. These hydrogel phases were found to be stationary for the duration of the tracking experiments. Images of the structure of these phases were obtained using cryo-SEM, shown in Fig. 4, these agree with the diffusive data showing a more heterogenous structure for the low and medium viscous hydrogels, with estimated pore sizes ranging from 100 nm to 1.5 µm, when compared to the high viscous presenting a more homogeneous and compact structure. Moreover, from the obtained micrographs medium viscous hydrogel seems to be the closest to mimicking vitreous humour (porcine) at a morphological level.

Following this preliminary investigation, a more detailed study was performed in which at least 30 randomly-selected nanoparticles were tracked in each sample with the aim of understanding the distribution of nanoparticle behavior in the hydrogels. Since the charge of the nanoparticles had been demonstrated to have no influence on their diffusion under these conditions, only citrate-capped gold nanoparticles with diameters of 50 nm, 100 nm and 200 nm were tested to investigate the effect of particle size on diffusion behavior in the hydrogels. These sizes were chosen because they are clinically relevant to back-of-the-eye drug delivery: for example, a study by Sakurai et al. found that, after 2 months of an intravitreal injection, 2 µm nanospheres remained in the vitreous, but 200 nm and 50 nm particles were able to reach the retina^38^. Coglitore et al.^39^ found that the diffusion behaviour of nanoparticles of this order of size in simple fluids was independent of particle density, but different to those of larger particles so the behaviour observed in this study is likely specific to nanoparticles of diameter 200 nm or less.

**Figure 5:**
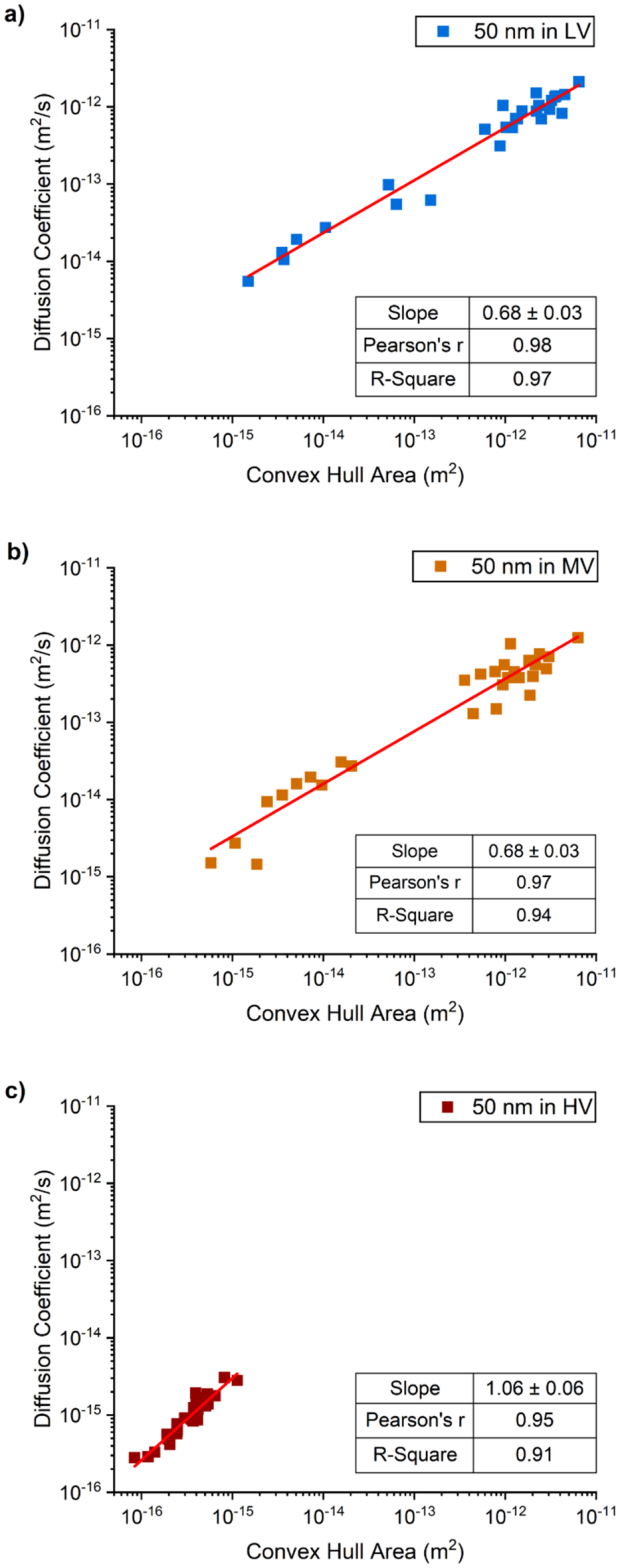
Correlation between the diffusion coefficient values of 50 nm diameter gold nanoparticles and the area of the convex hull of each nanoparticle trail of motion, in the (**a**) low viscous (blue); (**b**) medium viscous (orange); and (**c**) high viscous(red) hydrogels.

**Figure 6:**
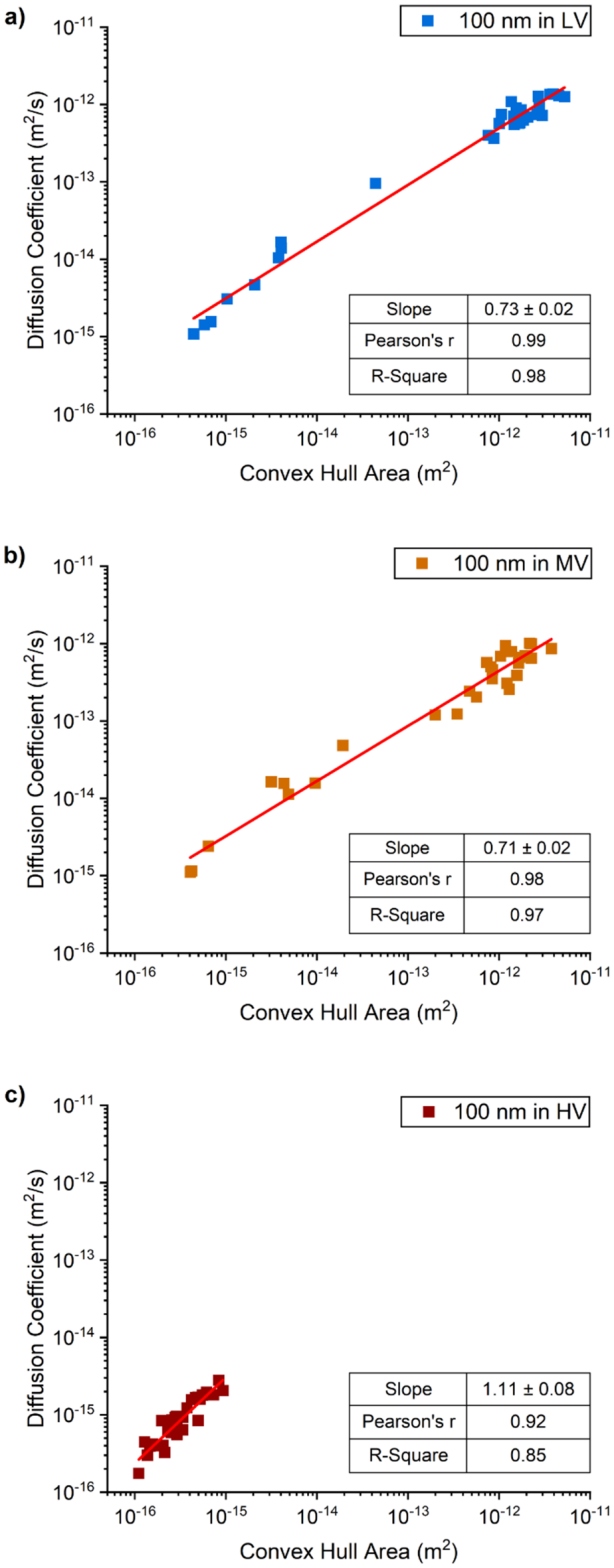
Correlation between the diffusion coefficient values of 100 nm diameter gold nanoparticles and the area of the convex hull of each nanoparticle trail of motion, in the (**a**) low viscous (blue); (**b**) medium viscous (orange); and (**c**) high viscous(red) hydrogels.

**Figure 7:**
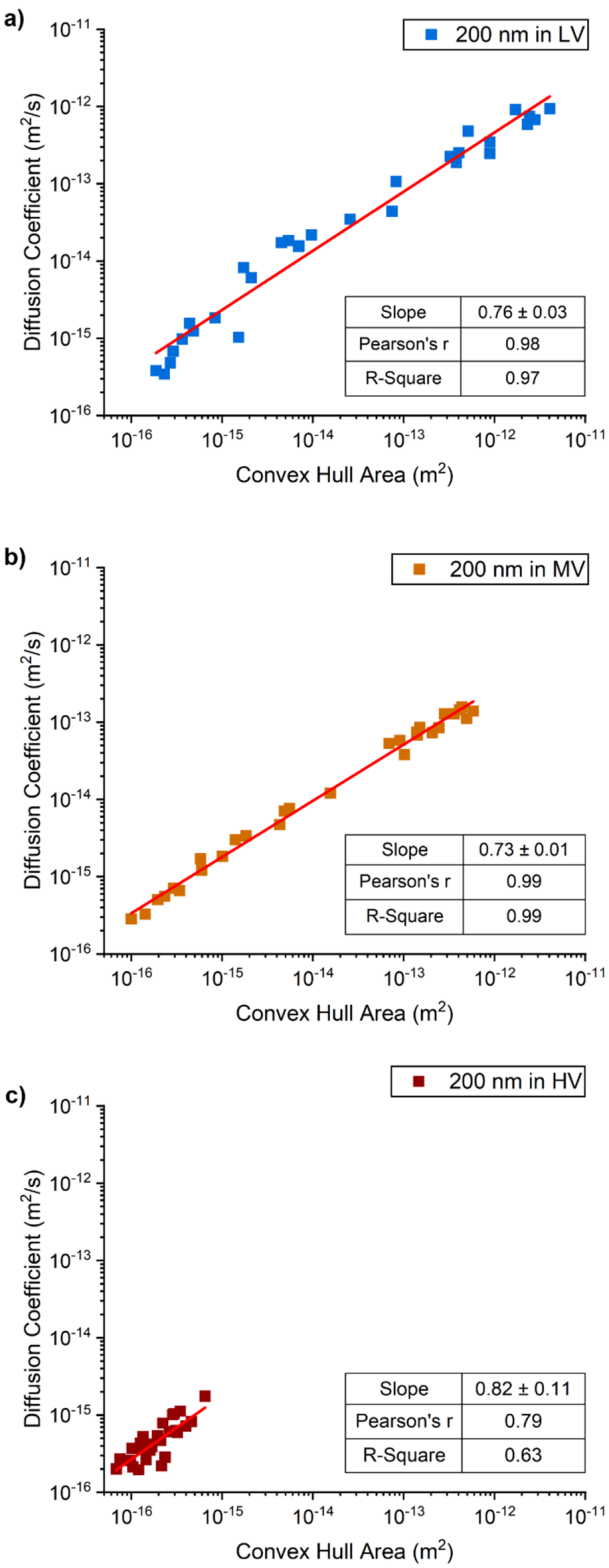
Correlation between the diffusion coefficient values of 200 nm diameter gold nanoparticles and the area of the convex hull of each nanoparticle trail of motion, in the (**a**) low viscous (blue); (**b**) medium viscous (orange); and (**c**) high viscous(red) hydrogels.

The values of the diffusion coefficient are shown in Figs. 5, 6, 7 as a function of the area of the convex hull enclosing the path for each tracked particle for the three hydrogels with low, medium and high viscosity. In each case a linear regression line was fitted to the data. The results confirm that the nanoparticles diffuse faster in gels with higher water content probably because there is less polymeric matrix (gel) to constrain their movement. This is consistent with earlier work in which it was observed that the diffusion coefficient of nanoparticles was an inverse logarithmic function of the viscosity of the fluid^39^.

Diffusion values in the high viscosity hydrogel were similar for all diameters of nanoparticles, with diffusion values in the range of 10^–15^ m^2^/s and lower, while the areas of the corresponding convex hulls were all in the range of 10^–15^ to 10^–16^ m^2^, suggesting that nanoparticle size did not affect nanoparticle diffusion in the hydrogel and that the motion of all nanoparticles was hindered by the polymeric matrix (red squares in Figs. 5, 6, 7). The values of correlation coefficient at this level of viscosity are in the range R^2^ = 0.9 to 0.65, decreasing with increasing particle diameter, implying that the correlation between speed and area enclosed by the path decreases with particle diameter.

However, the behavior of the nanoparticles in the low and medium viscosity hydrogels was found to be size dependent, with the range of values for both diffusion coefficient and convex hull area remaining approximately constant but reducing in absolute value with increasing particle diameter which implies that larger particles move more slowly and cover a smaller area. The correlation between the diffusion coefficient and the area of the convex hulls is positive and close to one (R^2^ = 0.94–0.99) for both the medium and low viscosity hydrogels. However, the distribution of the data points in the graphs (Figs. 5, 6, 7) for these hydrogels varies with particle diameter from an approximately uniform distribution for the 200 nm diameter particles to a bimodal distribution for the 100 nm and 50 nm diameter particles with a tendency for more data points at the higher end of the range, with diffusive values close to those in water. The bimodal distribution is perhaps more evident for the data from medium viscosity hydrogel (graph b) in Figs. 5, 6, 7. It is likely that the high viscosity hydrogel was relatively homogeneous with a uniform distribution of the polymeric matrix that inhibited the motion of all of nanoparticles; whereas the medium and low viscosity hydrogels were progressively less homogeneous with zones of low-density polymeric matrix that allowed relatively uninhibited motion and zones of high-density matrix that inhibited motion. An idealized structure of this nature would generate the bimodal distributions seen in Figs. 5, 6, 7 with the bimodality likely more pronounced in the medium viscosity hydrogel.

These results agree with reported bimodal distribution of nanoparticles through ex vivo porcine vitreous humour^40^. However, Tavakoli et al. found that nanoparticle charge (with similar ranges of surface charge for cationic and anionic NPs) had an effect when tracked in ex vivo vitreous humour, although their working NP concentration was at least one order of magnitude higher than the one used in this study. This makes the agar-hyaluronic acid-based hydrogels good in vitro vitreous substitutes to investigate the effect of nanoparticle size. Here, though, the conditions failed to predict charge-chemical effects, probably due to the neutral nature of the hydrogels and the low nanoparticle concentrations. Nevertheless, further efforts should be made to better mimic the vitreous humour for in vitro models; low viscous and medium viscous hydrogels seem to cause a similar NP diffusive behaviour to the observed in porcine ex vivo vitreous humour, whereas the high viscous hydrogel presents a more homogeneous and dense matrix profile, which is less representative of vitreous humour.

The characterized heterogenicity of the hydrogels based on their micro and nanoscale features (through diffusion of nanoparticles and cryo-SEM micrographs), suggests that the mesh size is not consistent throughout the hydrogels, affecting the nanoparticle diffusion in a size-dependent manner. These features are believed to be consistent and reproducible.

The results demonstrated that label-free, real-time tracking of particles provides a powerful tool for characterizing the dynamics of nanoparticles interactions in heterogeneous polymeric hydrogels and to evaluate the degree to which nanoparticle size affects their behavior within the hydrogels. The resultant understanding should inform the development of nanoparticle- based drug delivery using in vitro models when combined with the findings of previous work that reported nanoparticle diffusion to be strongly affected by electrostatic and van der Waals forces, and controlled by the ionic strength of the media^27^ as well as by the impact of the protein corona formation on the diffusion of nanoparticles^40^.

## Conclusions

The heterogenous nature of agar-hyaluronic acid hydrogels, which have been previously validated as in vitro substitutes for vitreous humour, have been observed and quantified using a standard optical microscope set up to generate near-coherent light and reveal optical signatures known as caustics. The caustics generated by nanoparticles have been used in a label-free tracking technique to obtain experimental values of their diffusion coefficient in various local environments and for a range of particle diameters. It was found that the charge on the particles did not influence their diffusion through the hydrogels; however, both the diameter of the particles and the structure of the hydrogel affected the diffusion characteristics of the particles. Nanoparticles with diameters of 50, 100 and 200 nm moved progressively more slowly and within a smaller area. High viscosity hydrogels were observed to generate consistent behaviour of the nanoparticles implying a large homogeneous structure of polymeric matrix, or gel that uniformly inhibited particle motion, whereas medium and low viscosity hydrogels generated bimodal distributions of particle diffusion coefficients as functions of the area enclosed by the particle’s path suggesting a heterogeneous hydrogel with zones of low and high density polymeric matrix in which the particles move more or less freely, respectively.

The results indicate that the single-particle label-free nanoparticle tracking technique is a powerful tool to characterise the diffusion and transport of nanoparticles in heterogeneous hydrogels that can be used as in vitro substitutes for vitreous humour. Hence, this technique has the potential to become an indispensable method in the development of pre-clinical models to optimise nanoparticle-based drug delivery systems for the treatment of retinal diseases, and to better characterise their pharmacokinetics profiles. These advances could help elucidate the variables that influence the diffusive behavior of nanoparticles leading to the optimization of drug delivery to the retina.

### Supplementary Information

Supplementary Information.

## Data Availability

The datasets generated during and/or analysed during the current study are available in the [DataCat: The Research Data Catalogue] repository, [https://doi.org/10.17638/datacat.liverpool.ac.uk%2F2603].
