# Supplementary material for: Diffusion of nanoparticles in heterogeneous hydrogels as vitreous humour in vitro substitutes
Source: Sci Rep. 2024 Jul 29;14:17441. doi: 10.1038/s41598-024-68267-0 (PMC11286744; doi:10.1038/s41598-024-68267-0)
Supplement: Supplementary file 1 — Supplementary Information. [file 41598_2024_68267_MOESM1_ESM.docx]

**Diffusion of Nanoparticles in Heterogeneous Hydrogels as Vitreous Humour *in vitro* Substitutes**

Moira Lorenzo Lopez^1,2 *^, Victoria R. Kearns^2^, Judith M. Curran^1^ and Eann A. Patterson^1^.

^1^School of Engineering, University of Liverpool, Liverpool L69 3BX, UK.

^2^Department of Eye and Vision Science, University of Liverpool, Liverpool L7 8TX, UK.

*M.Lorenzo-Lopez@liverpool.ac.uk

Supplementary information:

The rheological properties of the synthesised agar-hyaluronic acid hydrogels were characterized using an Anton Paar Modular Compact Rheometer (MCR 302) with a stationary lower plate. Due to the gel-like nature of the vitreous humour and the hydrogels, a parallel measuring geometry 40 mm in diameter with a rough surface was used. The temperature was set at 34˚C and the samples were incubated for 10 minutes to ensure homogeneous temperature. To eliminate solvent loss during the analysis of the samples, a solvent trap was used to create a thermally stable vapour barrier.

First, an oscillatory frequency sweep was performed to ensure correct synthesis comparing the results with those obtained by Thakur et al.^1^, and to compare to vitreous humour extracted from porcine eyes, the results are shown in Fig. S1, as a mean of 3 tests, where the error bars represent the standard deviation. These sweeps were performed at angular frequency values ranging from 0.1 to 100 rad s^-1^ and a constant shear strain of 3%.

Second, we performed a viscosity shear rate sweep, with shear rate values ranging from 0.01 to 100 s^-1^. Viscosity values of 3 samples (error bars representing the standard deviation from the mean) were analysed and fitted with the Carreau-Yasuda model to obtain static values of viscosity Fig. S2. (used as the bulk viscosity values for the hydrogels in the tracking process).

**Supplementary Figure 1**: Oscillatory frequency sweep of hydrogels and porcine vitreous humour at 34°C. The storage (G’) and Loss (G’’) modulus of high viscous (HV, red), medium viscous (MV, orange), low viscous (LV, blue) hydrogels, and porcine vitreous humour (VH, green) are compared. G’ and G’’ SD values over 40 rad/s are excluded due to poor resistance to high frequencies.

**Supplementary Figure 2**: Agar-hyaluronic acid hydrogels (LV- low viscous; MV-medium viscous; HV-high viscous) viscosity values measured at 34°C, fitted to the Carreau- Yasuda model to obtain static values of viscosity.

**Supplementary Table 1:** Gold nanoparticles characteristics.

| Hydrodynamic diameter (nm) | Zeta potential (mV) | Concentration in A-HA hydrogel (mg/mL) |
| --- | --- | --- |
| 50 | - 34.5 | 5x10^-4^ |
| 100 - cITRATE | - 32.7 | 5x10^-4^ |
| 100 - bpei | 28.7 | 5x10^-4^ |
| 200 | -31 | 5x10^-4^ |

Link to videos of nanoparticles in different media:

<https://doi.org/10.17638/datacat.liverpool.ac.uk%2F2603>

Reference:

1. Thakur, S. S., Shenoy, S. K., Suk, J. S., Hanes, J. S. & Rupenthal, I. D. Validation of hyaluronic acid-agar-based hydrogels as vitreous humor mimetics for in vitro drug and particle migration evaluations. *Eur. J. Pharm. Biopharm.* **148**, 118–125 (2020).
